# Supplementary figures and images for: Asexual reproduction of a few genotypes favored the invasion of the cereal aphid Rhopalosiphum padi in Chile
Source: PeerJ. 2019 Jul 26;7:e7366. doi: 10.7717/peerj.7366 (PMC6662566; doi:10.7717/peerj.7366)

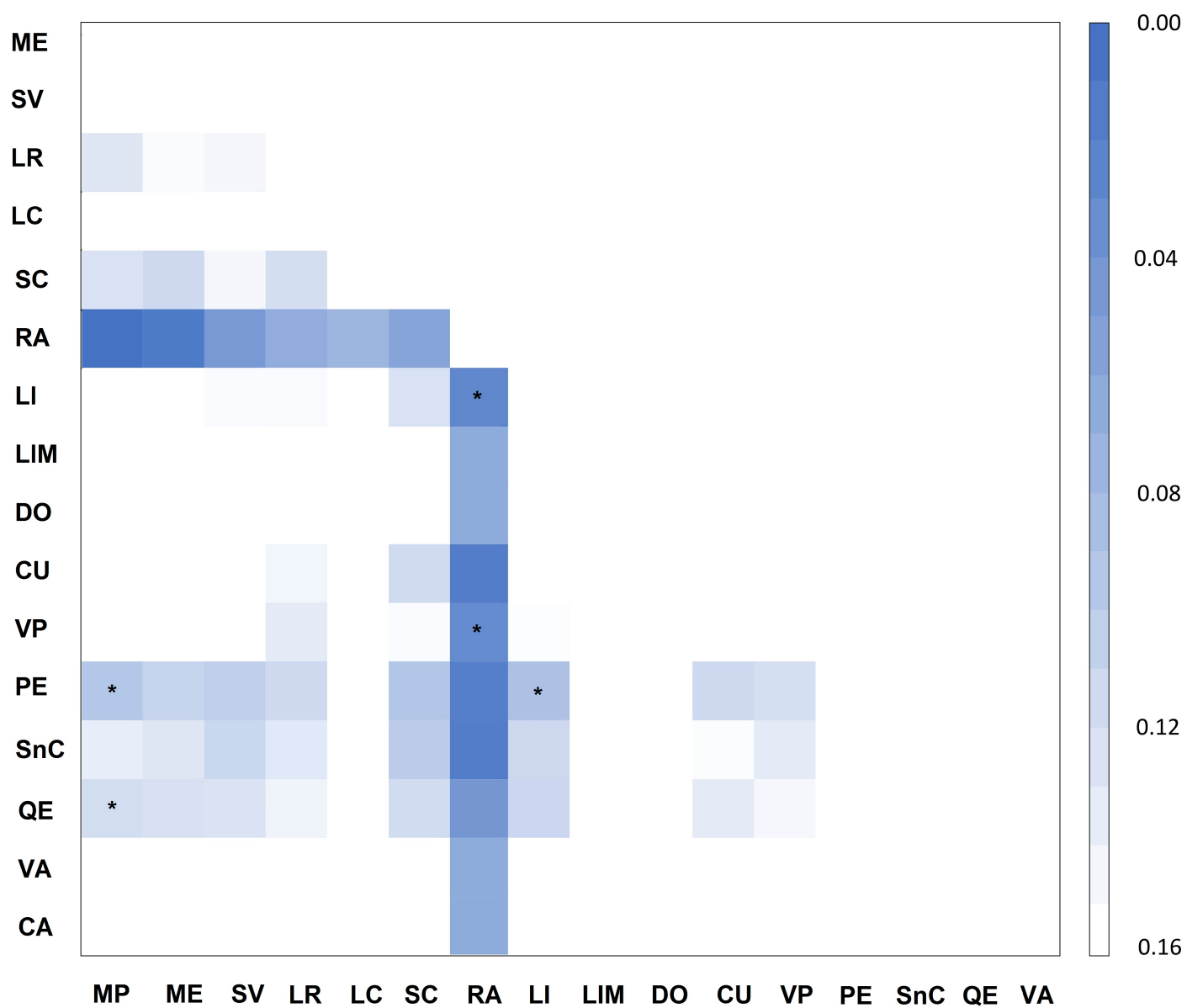

Supplement: Supplemental Information 1 — The range of colours from blue to white indicates decreasing pairwise genetic differentiation. Asterisks in bold are indicated the P-values that are below the significance value 0.000368, obtained after applying Bonferroni’s correction for multiple tests. [file peerj-07-7366-s001.pdf]

$$\text{DeltaK} = \text{mean}(|L''(K)|) / \text{sd}(L(K))$$

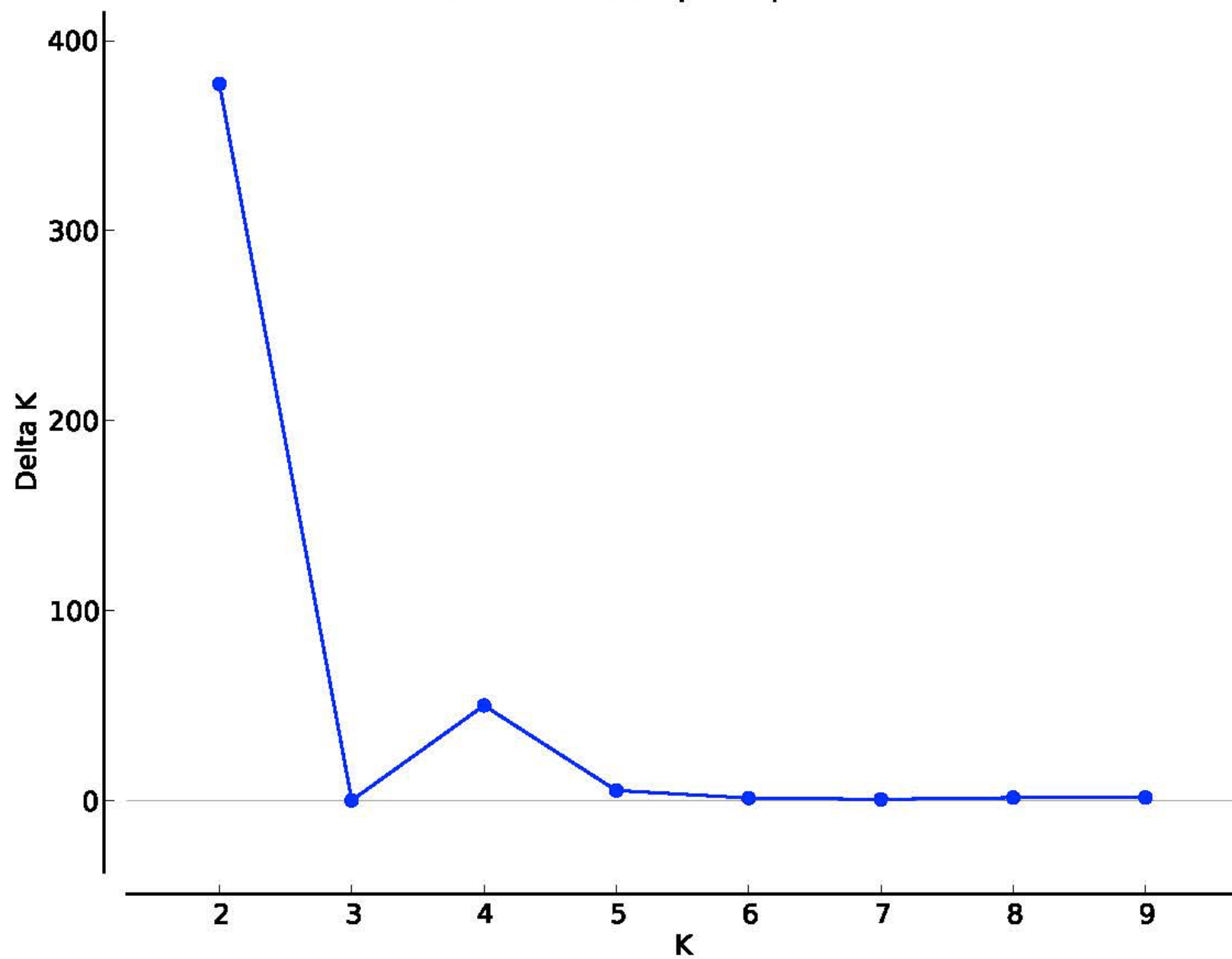

Supplement: Supplemental Information 2 [file peerj-07-7366-s002.pdf]
