# Supplementary material for: Asexual reproduction of a few genotypes favored the invasion of the cereal aphid Rhopalosiphum padi in Chile
Source: PeerJ. 2019 Jul 26;7:e7366. doi: 10.7717/peerj.7366 (PMC6662566; doi:10.7717/peerj.7366)
Supplement: Supplemental Information 5 — Locus name, central repeat motif, locus-specific hybridization temperature (Ta), and the number of alleles amplified with in each microsatellite locus. [file peerj-07-7366-s005.docx]

**Table S1. Characteristics of the six microsatellite loci used to study of *Rhopalosiphum padi***: locus name, central repeat motif, locus-specific hybridization temperature (Ta), and the number of alleles amplified within each microsatellite locus.

| **Locus** | **Repeat motif** | **Primer sequence** | **Ta** | **N. of alleles** | **Size range** |
| --- | --- | --- | --- | --- | --- |
| *R5.10* | (CA)_3_AC(AG)_6_(ATT)_3_…(GA)_15_ | F:CCGACTAAGCTTAATATTGTTTG  R:CGGTTCGGAGAACATAAGAG | 60 | 8 | 256-274 |
| *R2.73* | (GT)_25_CG(ATT)_4_ | F:GTCGTTTCTGGTCAGCGGCC  R:GTCGTTTCTGGTCAGCGGCC | 60 | 6 | 262-285 |
| *R5.29.b* | (AC)_13_ | F:CATGCGTGTGTCCCTTTTAAC  R:GATGGACGAGGGGACAC | 60 | 17 | 161.216 |
| *R3.171* | (AT)_2_T(AT)_9_…(AC)_11_ | F:TGTACATCGTAAGACGTAAAACGAC  R:CAAAGCAATACCTCATAACG | 60 | 15 | 214-252 |
| *R5.138* | (CA)_35_ | F:TATACACGCTCGCGCTTACG  R:CCGAGCACGAATTATTCC | 60 | 20 | 211-287 |
| *R5.50* | (AC)_22_ | F:TGTTACGCGGAGTGTGTAGG  R:CCACAGAGCGTTGTGCATC | 60 | 25 | 297-403 |
