# Supplementary material for: Asexual reproduction of a few genotypes favored the invasion of the cereal aphid Rhopalosiphum padi in Chile
Source: PeerJ. 2019 Jul 26;7:e7366. doi: 10.7717/peerj.7366 (PMC6662566; doi:10.7717/peerj.7366)
Supplement: Supplemental Information 6 — P-value for each locus pair across all populations (Fisher’s method). Analyses were performed on dataset without repeated genotypes (one single copy per MLG). Asterisks indicates the P-values that are below the significance value 0.00033, obtained after applying Bonferroni’s correction for multiple tests. [file peerj-07-7366-s006.docx]

**Table S2. Linkage disequilibrium analysis of *Rhopalosiphum padi* populations in central Chile**. *P*-value for each locus pair across all populations (Fisher's method). Analyses were performed in the dataset without repeated genotypes (one single copy per MLG). Asterisks indicates the *P*-values that are below the significance value 0.0033, obtained after applying Bonferroni’s correction for multiple tests.

| **Locus pair** | | **χ^2^** | **df** | ***P*-Value** |
| --- | --- | --- | --- | --- |
| *R550* | *R5138* | 38.32 | 16 | 0.0015 * |
| *R550* | *R3171* | 27.78 | 16 | 0.0395 |
| *R5138* | *R3171* | 22.51 | 16 | 0.1221 |
| *R550* | *R273* | 28.62 | 16 | 0.0257 |
| *R5138* | *R273* | 42.14 | 18 | 0.0010 * |
| *R3171* | *R273* | 34.24 | 12 | 0.0006 * |
| *R550* | *R510* | 13.19 | 18 | 0.7805 |
| *R5138* | *R510* | 11.90 | 20 | 0.9196 |
| *R3171* | *R510* | 11.08 | 20 | 0.9427 |
| *R273* | *R510* | 26.75 | 18 | 0.0824 |
| *R550* | *R529b* | 28.12 | 16 | 0.0279 |
| *R5138* | *R529b* | 43.31 | 18 | 0.0007 * |
| *R3171* | *R529b* | 33.35 | 14 | 0.0029 * |
| *R273* | *R529b* | 54.23 | 18 | 0.0001 * |
| *R510* | *R529b* | 31.27 | 18 | 0.0209 |
