# Supplementary material for: Asexual reproduction of a few genotypes favored the invasion of the cereal aphid Rhopalosiphum padi in Chile
Source: PeerJ. 2019 Jul 26;7:e7366. doi: 10.7717/peerj.7366 (PMC6662566; doi:10.7717/peerj.7366)
Supplement: Supplemental Information 7 — Number of nymphs, apterous, alates and population growth of rate after 14 days of infestation under controlled conditions. Different letters within columns represent statistically significant differences according to Tukey’s HSD test (P < 0.05). [file peerj-07-7366-s007.docx]

**Table S3**. **Reproductive performance of five clonal lineages of the Rp1 genotype of *Rhopalosiphum padi* tested on four hosts.** The number of nymphs, apterous, alates and population growth of rate after 14 days of infestation under controlled conditions. Different letters within columns represent statistically significant differences according to Tukey's HSD test (*P* < 0.05).

|  |  |  | **Nymphs** | | | | **Adults** | | | | **Alates** | | | | **PGR** | | | | | | | |  |
| --- | --- | --- | --- | --- | --- | --- | --- | --- | --- | --- | --- | --- | --- | --- | --- | --- | --- | --- | --- | --- | --- | --- | --- |
| **Clone** | **Host** | **N** | **Mean** |  | **SD** |  | **Mean** |  | **SD** |  | **Mean** |  | **SD** |  | **Mean** | |  | | **SD** | |  | |  |
| Rp1-Licantén | *T. aestivum* | 5 | 198.00 | ± | 91.61 | def | 16.20 | ± | 9.73 | a | 20.60 | ± | 16.38 | b | 0.24 | | ± | | 0.02 | | abc | |  |
|  | *H. vulgare* | 5 | 214.40 | ± | 67.73 | gh | 33.00 | ± | 9.19 | c | 18.40 | ± | 15.13 | b | | 0.25 | | ± | | 0.01 | | abc | |
|  | *A. sativa* | 5 | 296.00 | ± | 97.79 | h | 49.40 | ± | 29.42 | b | 7.80 | ± | 5.85 | a | | 0.26 | | ± | | 0.02 | | abc | |
|  | *T. turgidum* | 5 | 289.60 | ± | 53.25 | j | 15.60 | ± | 4.93 | a | 16.80 | ± | 3.27 | b | | 0.26 | | ± | | 0.01 | | c | |
| Rp1-Docamávida | *T. aestivum* | 5 | 195.40 | ± | 102.10 | cd | 25.80 | ± | 11.80 | b | 23.60 | ± | 11.08 | b | | 0.24 | | ± | | 0.03 | | abc | |
|  | *H. vulgare* | 5 | 239.80 | ± | 115.68 | def | 15.40 | ± | 5.37 | a | 20.60 | ± | 12.68 | b | | 0.24 | | ± | | 0.03 | | abc | |
|  | *A. sativa* | 5 | 243.80 | ± | 101.75 | efg | 27.20 | ± | 5.85 | b | 7.60 | ± | 4.83 | a | | 0.25 | | ± | | 0.02 | | abc | |
|  | *T. turgidum* | 5 | 374.80 | ± | 135.45 | i | 33.60 | ± | 4.56 | b | 27.80 | ± | 28.29 | b | | 0.28 | | ± | | 0.02 | | cb | |
| Rp1-Limávida | *T. aestivum* | 5 | 102.80 | ± | 65.72 | a | 16.40 | ± | 15.71 | a | 2.80 | ± | 2.05 | a | 0.18 | | ± | | 0.05 | | a | |  |
|  | *H. vulgare* | 5 | 147.00 | ± | 94.71 | bc | 22.20 | ± | 14.81 | ab | 3.00 | ± | 3.08 | a | 0.21 | | ± | | 0.05 | | abc | |  |
|  | *A. sativa* | 5 | 128.80 | ± | 102.07 | b | 21.00 | ± | 9.46 | ab | 2.60 | ± | 3.78 | a | 0.19 | | ± | | 0.06 | | ba | |  |
|  | *T. turgidum* | 5 | 288.40 | ± | 96.23 | i | 27.20 | ± | 17.08 | b | 3.80 | ± | 4.92 | a | 0.26 | | ± | | 0.02 | | bc | |  |
| Rp1-Villa Alegre | *T. aestivum* | 5 | 174.80 | ± | 43.31 | def | 40.80 | ± | 20.22 | c | 15.80 | ± | 6.98 | a | 0.24 | | ± | | 0.02 | | abc | |  |
|  | *H. vulgare* | 5 | 190.40 | ± | 24.34 | fgh | 12.20 | ± | 4.71 | a | 13.80 | ± | 9.52 | a | 0.23 | | ± | | 0.01 | | abc | |  |
|  | *A. sativa* | 5 | 208.00 | ± | 59.47 | i | 23.00 | ± | 4.64 | b | 18.80 | ± | 9.73 | a | 0.24 | | ± | | 0.02 | | cb | |  |
|  | *T. turgidum* | 5 | 325.80 | ± | 126.03 | i | 20.00 | ± | 10.44 | ab | 18.80 | ± | 15.64 | a | 0.26 | | ± | | 0.02 | | cb | |  |
| Rp1-Cato | *T. aestivum* | 5 | 181.00 | ± | 27.96 | de | 32.00 | ± | 5.96 | b | 12.80 | ± | 8.64 | ab | 0.24 | | ± | | 0.01 | | abc | |  |
|  | *H. vulgare* | 5 | 202.20 | ± | 45.19 | def | 16.80 | ± | 8.64 | a | 12.80 | ± | 7.69 | ab | 0.24 | | ± | | 0.01 | | abc | |  |
|  | *A. sativa* | 5 | 243.00 | ± | 97.94 | h | 19.20 | ± | 9.20 | a | 7.40 | ± | 5.59 | a | 0.25 | | ± | | 0.02 | | abc | |  |
|  | *T. turgidum* | 5 | 428.00 | ± | 116.52 | k | 57.60 | ± | 25.30 | c | 17.20 | ± | 10.52 | b | 0.29 | | ± | | 0.02 | | c | |  |
